# Supplementary material for: Altered stability of nuclear lamin-B marks the onset of aging in male Drosophila
Source: PLoS One. 2022 Mar 24;17(3):e0265223. doi: 10.1371/journal.pone.0265223 (PMC8947137; doi:10.1371/journal.pone.0265223)

**Supporting Information**

**Altered stability of nuclear lamin-B marks the onset of aging in male *Drosophila***

Wei-Qi Lin, Zhen-Kai Ngian, Tong-Wey Koh, Chin-Tong Ong

**Checklist**

1) Supporting S1 to S4 Figures with legends.

2) Supporting Experimental Procedures

3) Authors contribution

4) Conflict of interest

5) Data availability: S1 to S7 Tables.

https://doi.org/10.6084/m9.figshare.19307036

6) Supporting References

7) S1 Table

8) Original uncropped immunoblot images

## 1) Supporting Figures

**Figure S1**

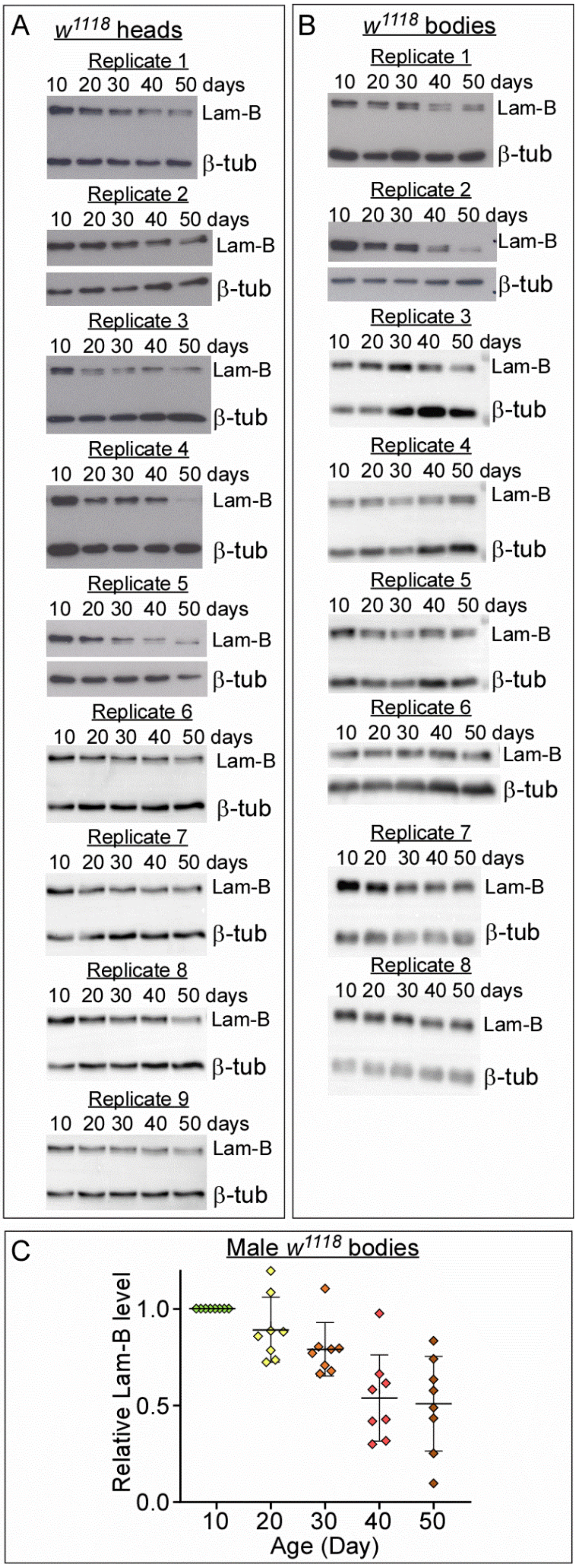

**S1 Fig. Gradual decline in the level of lamin-B protein in young adult male flies.**

(A-B) Immunoblot of the head and bodies lysates harvested from male $w^{1118}$ at different ages. Each biological replicate contains heads or bodies harvested from five adult flies.

(C) Quantification of the body lysates harvested from male $w^{1118}$ at different time-points. Each biological replicate (diamond) contained five bodies. Data are presented as level of nuclear lamin-B (normalized to β-tubulin) relative to Day10 flies ± SD.

Figure S2

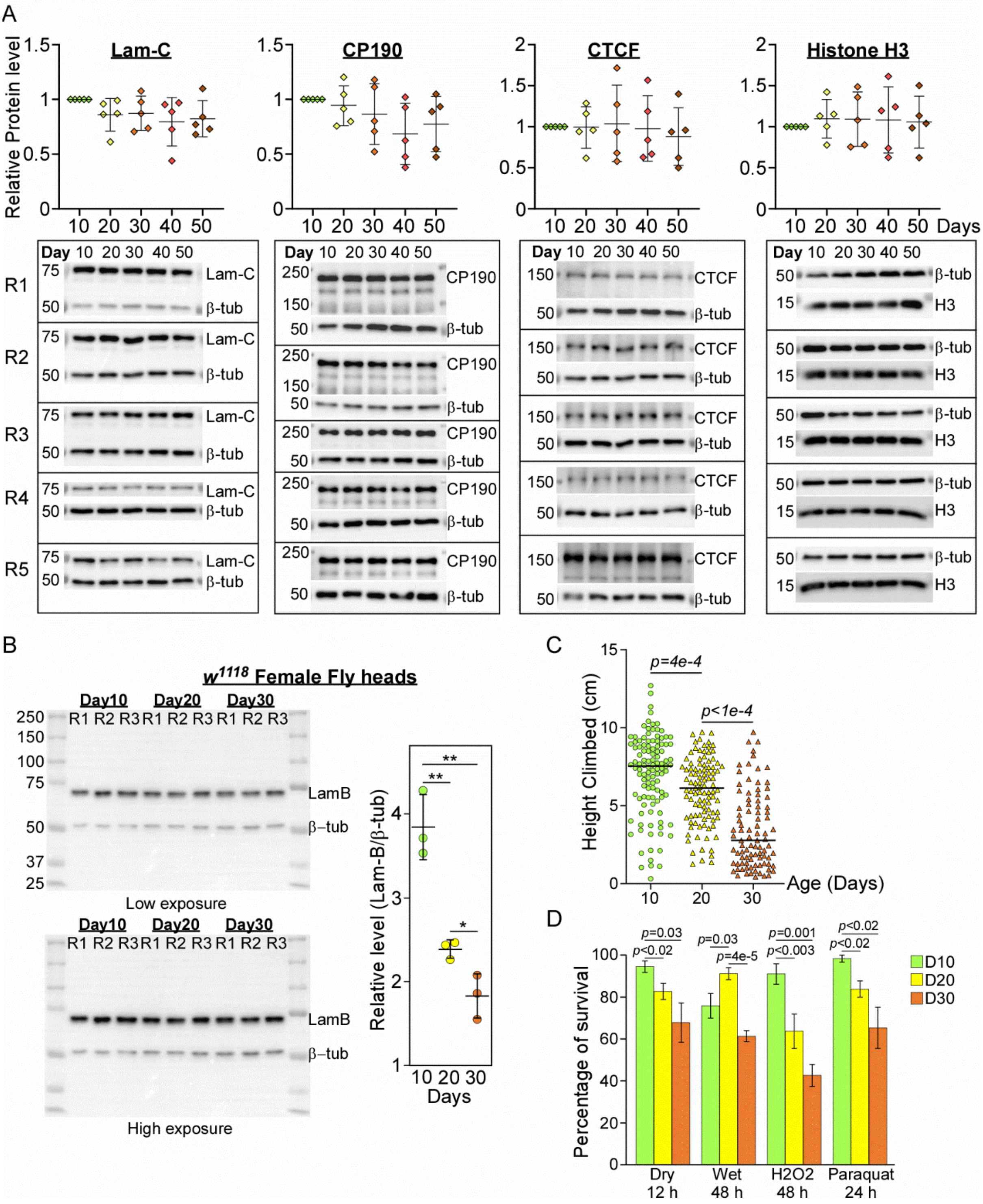


**S2 Fig. Patterns of other nuclear proteins during aging and results of female flies.**

(A) (Top) Quantification of the head lysates harvested from male $w^{1118}$ at different time-points. Each biological replicate (diamond) contained five heads. Data are presented as level of protein (normalized to β-tubulin) relative to Day10 flies ± SD. Refer to Table S2 for $p$-value. (Bottom) Immunoblot of the head lysates harvested from male $w^{1118}$ at different ages with antibodies

against nuclear lamin-C, architectural protein CP190 and CTCF, histone H3 and β-tubulin (as loading control). Each biological replicate (R1-R5) contains five heads.

(B) (Left) Immunoblot of the head lysates harvested from female $w^{1118}$ at different ages with antibodies against nuclear lamin-B and β-tubulin. Each biological replicate (R1-R5) contains five heads. (Right) Quantification of the head lysates harvested from female $w^{1118}$ at different time-points. Each biological replicate (circle) contains five heads. Data are presented as level of lamin-B normalized to β-tubulin ± SD. * $p < 0.03$, ** $p < 0.005$. 2-tailed *t*-test.

(C) Locomotor activity of $w^{1118}$ females at different ages. Each triangle represents a fly and bar denotes mean. Mann-Whitney test.

(D) Stress tests where female flies were cultured in empty vial (dry), 1% agarose (wet), food with 1% $H_2O_2$ or 10 mM Paraquat.

**Figure S3**

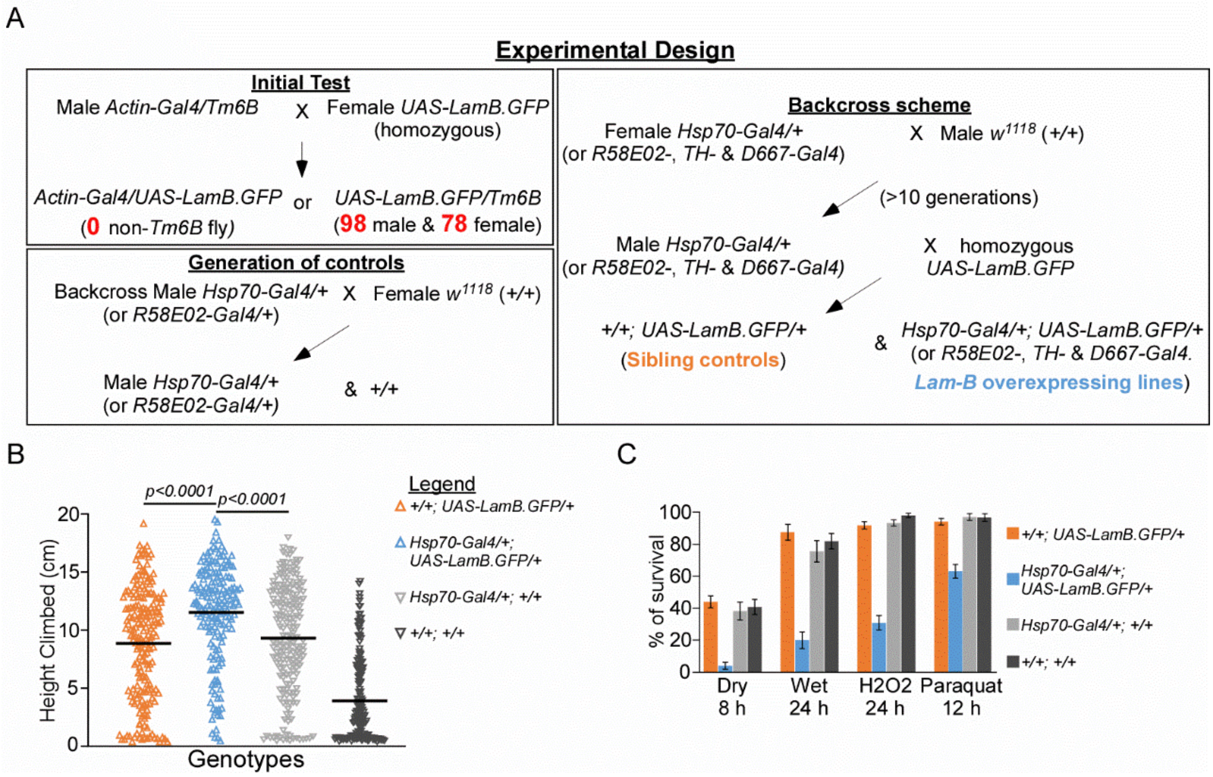


**S3 Fig. Experimental design and characterization of *Hsp70-Gal4/UAS-LamB-GFP* line**

(A) Schematic of genetic crosses. (Top left) Initial crossing of *Actin-Gal4* with homozygous *UAS-LamB.GFP* line. (Bottom left) Mating of backcrossed heterozygous *Gal4/+* males with $w^{1118}$ females to obtain *Gal4/+* and +/+ controls siblings for stress test, lifespan and climbing assays. (Right) Female virgins of different *Gal4/+* lines were backcrossed to $w^{1118}$ males for at least 5 to 10 (for *Hsp70* and *R58E02*) generations. The resulted backcrossed heterozygous *Gal4/+* males were then mated with homozygous *UAS-LamB.GFP* line to obtain different *Gal4/UAS-LamB.GFP* flies and control siblings *UAS-LamB.GFP/+* for assays.

(B) Locomotor activity of Day 30 non-heat-shocked *Hsp70-Gal4/+; UAS-LamB.GFP/+* males and their various control siblings. Each triangle represents one fly. Mann-Whitney test.

(C) Stress tests where male flies of different genotypes were cultured in empty vial (dry), 1% agarose (wet), food with 1% $H_2O_2$ or 10 mM Paraquat.

**Figure S4**

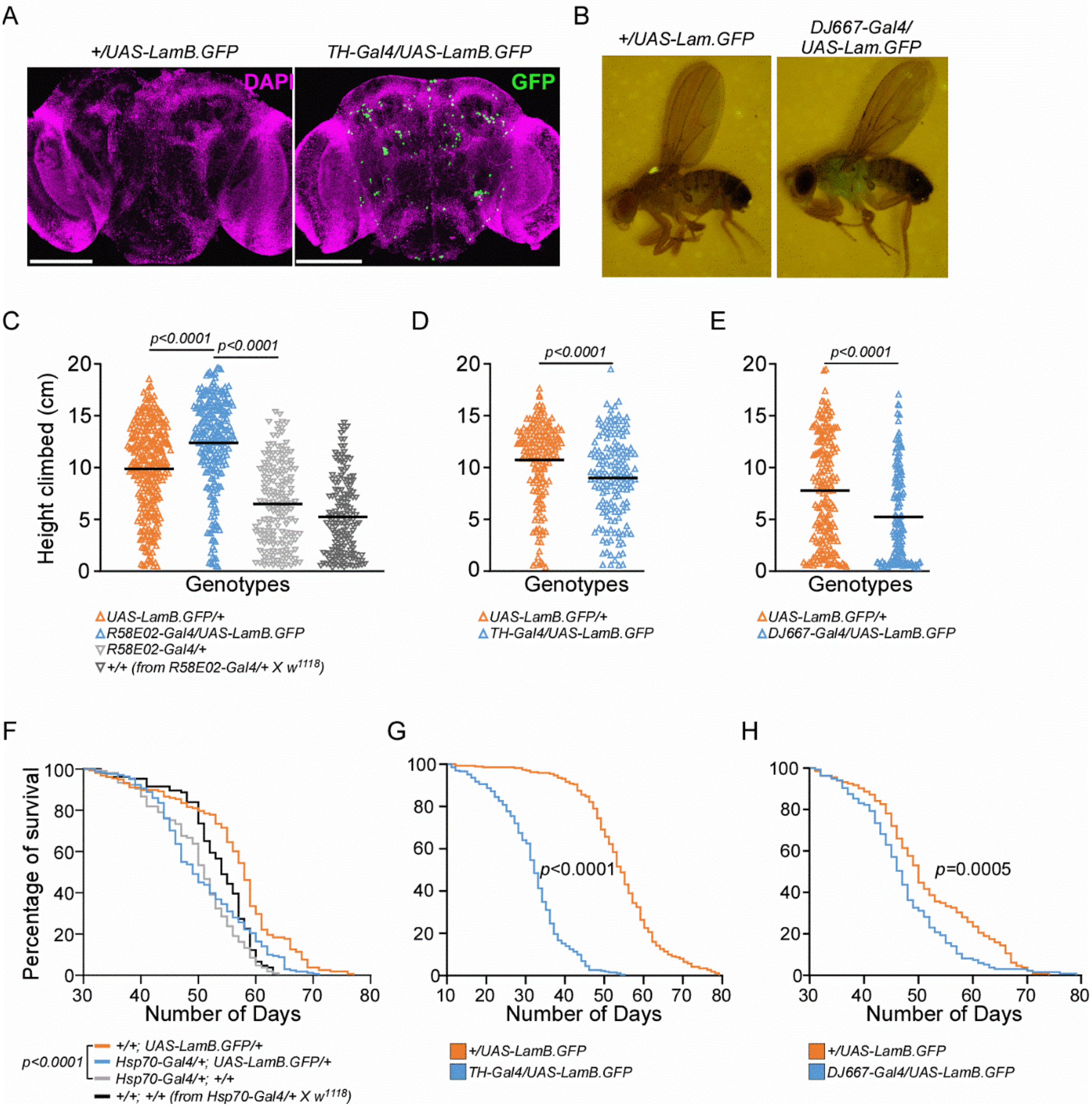


**S4 Fig. The impact of ectopic LamB-GFP expression on male fly is highly tissue-specific**

(A) Expression pattern of GFP-tagged *Lamin-B* in the brains of *TH-Gal4/UAS-LamB.GFP* fly and its control sibling. Scale bars represent 150 µm. (B) Images of *DJ667-Gal4/UAS-LamB.GFP* and its control sibling. (C-E) Locomotor activity of Day 30 control siblings and *lamin-B* overexpressing lines that were induced by different *Gal4* lines. Mann-Whitney test. (F-H) Lifespan assay of (F) non-heat-shocked *Hsp70-Gal4/+; UAS-LamB.GFP/+* flies, (G) *TH-Gal4/UAS-LamB.GFP* flies, (H) *DJ667-Gal4/UAS-LamB.GFP* flies and their control siblings. Log-rank test.

## 2) Supporting Experimental Procedure

**Fly strains and husbandry**

*Drosophila melanogaster* were cultured in standard fly media (6% cornmeal, 5% dextrose, 2.4% brewer's yeast, 0.8% agarose and 0.3% nipagin). Twenty male flies were housed in a cylindrical vial (height 9.5cm, diameter 2.5cm) at 25°C with 12-hour day/night cycle. Eclosed adults were considered as day 0 and vial was changed every two days until the appropriate experiments.

**Rapid Iterative Negative Geotaxis (RING) Assay**

Flies were transferred to fresh vials of food a day before the experiment. On the day of assay, 10-15 flies were transferred directly without carbon dioxide anaesthesia into glass measuring glass cylinder (Height 22 cm, Outer diameter 2.5 cm, capacity 50 ml, AS ONE) capped by a cotton plug. The flies were tapped to the bottom and picture was taken by digital camera after 6 sec to capture the distance climbed by the flies. This method was repeated until a sizeable population (at least 100 flies) was obtained for each condition. The height climbed for individual fly was displayed in a scatter plot and Mann-Whitney test (Prism) was performed to determine the statistical significance between the two genotypes or age groups.

**Whole-mount Immunostaining, Confocal Imaging, and Image Processing**

Five Day10 or 30 male $w^{1118}$ flies were processed each time. Heads removed from animals under light $CO_2$ anaesthesia were fixed (4% formaldehyde, 0.4% Triton-X, PBS) for 15 min at room temperature and washed five times in 0.4% PBST (0.4% Triton-X, PBS). Whole brains were dissected from the fixed heads in cold PBS and transferred immediately into cold fixative (same as above). The brains were fixed for 20 min at room temperature followed by 5 min wash in 0.4% PBST. After blocking in 5% NGS in 0.4% PBST, they were incubated with anti-lamin Dm0 (1:2000, DSHB, Cat#ADL67.10-c) and anti-tyrosine hydroxylase (TH) (1:2000, Novus Biological, Cat#NB300-109) over two nights at 4°C. The brains were subjected to five 0.4% PBST washes, followed by incubating with Alexa Fluor 594 anti-mouse IgG (1:500, Invitrogen, Cat#A11005) and FITC anti-rabbit IgG (1:500, Invitrogen, Cat#A16097) antibodies in the dark for two nights at 4°C. Brains were washed five times in 0.4% PBST and incubated with Hoechst 33258 dye (1:10000, Thermo Fisher, Cat#H21491) in the dark for 20 min at room temperature. After five 0.4% PBST washes, brains were mounted using VECTASHIELD Mounting Medium (Vector Laboratories, Cat#H-1000) with the anterior side orientated towards the coverslip. The experiments were repeated three times.

Images were taken using Olympus FV3000 inverted confocal system (Version 2.3.1.163) with 60x objective lens, x2.5 optical zoom and the following settings: DAPI (1% laser transmissivity and PMT Voltage of 450V), FITC (1% and 390V), Alexa Fluor 594 (1% and 525V) and sampling speed of 4.0 μsec/pixel. About 30 *z*-sections were captured at 0.3 μm apart to generate 1024 x 1024 3-dimensional (3D) images of TH-specific cells in the PAM regions in the left and right lobes of the brain. Images were analysed with Imaris software (Version 9.2.0). ImarisColoc was implemented to define TH and lamin-B double-positive cells. The intensity threshold for FITC and Alexa Fluor 594 channels was set at ≥ 1000; and the co-localization voxels at ≥ 40000. ImarisCell module was next implemented to determine individual cells and their signals intensity. The mean Lamin-B/DAPI (i.e. Hoechst 33258) of individual cells and the brains were calculated for Day10 and 30 age groups.

**RNA extraction and Quantitative RT-PCR**

Male $w^{1118}$ flies at Day10, 20, 30, and 50 were harvested. Total RNA was isolated from 20 fly heads using RNAzol® RT (Sigma, Cat#R4533). After DNase I treatment (Thermo Scientific, Cat# EN0521), the RNA was converted to cDNA using High-Capacity cDNA Reverse Transcription Kit (Applied Biosystems, Cat# 4368814). Quantitative real-time PCR was performed on the cDNA using Maxima SYBR Green/ROX qPCR Master Mix (Thermo Scientific, USA, Cat# K0222) with ABI 7900HT instrument (Applied Biosystems). The primers (5' to 3') used were as follow:

*Lamin-B (Lamin Dm0)*
F: AGCCAATGCCGACCTCAATG
R: GGTGATGTTCAAACGAGCCT
*Actin 5C*
F: GGCGCAGAGCAAGCGTGGTA
R: GGGTGCCACACGCAGCTCAT

**Immunoprecipitation (IP)**

IP was performed with ~200 $w^{1118}$ male flies harvested at Day10 and 30 given that this is the time-point where lamin-B in fly bodies showed significant reduction. Intact nuclei pellet was prepared as described in CETSA protocol and resuspended in 1 mL of cold RIPA buffer (10mM Tris pH8.0, 1mM EDTA, 1% Triton-X, 140 mM NaCl, 0.2% SDS, 0.2% sodium deoxycholate, 0.5mM DTT, phosphatase inhibitor cocktails 2 & 3 from Sigma; Leupeptin, Pepstatin A,

Aprotinin, and 0.5mM PMSF). The lysates were homogenized with 20 strokes of Pestle B (Sigma, Cat# D8938) and rocked on ice for 10 min. The lysates were then incubated with 12.5U/mL of benzonase nuclease (Sigma, Cat#E1014) in the presence of 1 mM of $CaCl_2$ and $MgCl_2$ for 1 hour at 4°C. After centrifugation at 15,000 x g for 10 min in cold, the nuclei-containing supernatant was pre-cleared with Sepharose™ protein-A beads (GE Healthcare, Cat#17-1279-01) conjugated to mouse IgG antibody. The concentration of pre-cleared lysates was measured with Pierce™ BCA (Thermo Scientific™, Cat#23225). 5% of pre-cleared lysate was kept as input while the remaining were split equally for IP using either 5µg of IgG or Lamin-B (DSHB, Cat#ADL67.10-s) antibodies bound to protein-A beads. After overnight IP, the beads were washed three times with cold RIPA buffer and centrifuged at 1,000 x rpm for 2 min. Proteins captured by antibodies and the 5% input were boiled in 2x and 6x Laemmli buffer respectively. IP was repeated 6 times and the relative level of 100 kD Lamin-B band purified from Day10 and 30 flies was calculated as described earlier.

## 3) Author Contributions

C.T.O. conceived and designed this study. W.Q.L, Z.K.N and C.T.O. conducted the experiments and analysed the data. C.T.O., W.Q.L and T.W.K. interpreted the results. W.Q.L. and C.T.O wrote the manuscript. W.Q.L., Z.K.N., C.T.O. and T.W.K. have reviewed and approved the final version of the manuscript.

## 4) Conflict of interest

The authors have no conflict of interest.

## 5) Data Availability

The data that support the findings of this study are available as follow:

https://doi.org/10.6084/m9.figshare.19307036

S2 Table. Time-course western analysis

S3 Table. Climbing (RING) assay of different genotypes

S4 Table. Stress test of different genotypes

S5 Table. Summary of immunofluorescence of PAM DNs

S6 Table. Lifespan assay

S7 Table. Western analysis of soluble CP190 and Lamin-B by CETSA

All the IF images were uploaded to the following link:
https://data.mendeley.com/datasets/rz4c9b29sm/1 (dx.doi.org/10.17632/rz4c9b29sm.1)

## 6) Supporting References

1. Jafari, R., Almqvist, H., Axelsson, H., Ignatushchenko, M., Lundback, T., Nordlund, P., and Martinez Molina, D. (2014). The cellular thermal shift assay for evaluating drug target interactions in cells. Nat Protoc *9*, 2100-2122.

2. Ong, C.T., Van Bortle, K., Ramos, E., and Corces, V.G. (2013). Poly(ADP-ribosyl)ation regulates insulator function and intrachromosomal interactions in Drosophila. Cell *155*, 148-159.

## 7) S1 Table. Summary of all statistical analyses

| Figure | Type of Assay | Condition 1 (Sample size) [Mean/Median] | Condition 2 (Sample size) [Mean/Median] | Statistical Test | *p*-value |
|---|---|---|---|---|---|
| 1A, S1A | Western of Lamin-B in fly heads | Day 10 (9 replicates; 5 heads/replicate) [Mean = 1.0] | Day 20 (9 replicates; 5 heads/replicate) [Mean = 0.65] | 2-tailed, paired *t*-test | < 0.0001 |
| S1B, S1C | Western of Lamin-B in fly bodies | Day 10 (8 replicates; 5 bodies/replicate) [Mean = 1.0] | Day 30 (8 replicates; 5 bodies/replicate) [Mean = 0.74] | 2-tailed, paired *t*-test | 0.003 |
| 1C | Climbing | Day 10 (n = 160) [Mean = 11.74 cm] | Day 20 (n= 153) [Mean = 6.01 cm] | Mann-Whitney | < 0.0001 |
| | Climbing | Day 10 (n = 160) [Mean = 11.74 cm] | Day 30 (n= 147) [Mean = 5.89 cm] | Mann-Whitney | < 0.0001 |
| 1D | 12 h Dry starvation | Day 10 (n = 100) [66% mean survival] | Day 20 (n= 100) [8% mean survival] | Mann-Whitney | < 0.0001 |
| | 48 h Wet starvation | Day 10 (n = 120) [53.3% mean survival] | Day 20 (n= 120) [17.5% mean survival] | Mann-Whitney | 0.0003 |
| | 48 h 1% $H_2O_2$ | Day 10 (n = 120) [94.2% mean survival] | Day 20 (n= 120) [50% mean survival] | Mann-Whitney | <0.0001 |
| | 24 h 10mM PQ | Day 10 (n = 99) [65.2% mean survival] | Day 20 (n= 99) [38.3% mean survival] | Unpaired *t*-test | 0.0017 |
| 2D | Climbing | *Hsp70-Gal4/+; UAS-LamB.GFP/+* (n = 190) [Mean = 11.45 cm] | *+/+; UAS-LamB.GFP/+* (n= 194) [Mean = 8.87 cm] | Mann-Whitney | < 0.0001 |
| 2E | 8 h Dry starvation | *Hsp70-Gal4/+; UAS-LamB.GFP/+* (n = 99) [4% mean survival] | *+/+; UAS-LamB.GFP/+* (n = 100) [44% mean survival] | Mann-Whitney | < 0.0001 |
| | 24 h Wet starvation | *Hsp70-Gal4/+; UAS-LamB.GFP/+* (n = 120) [20% mean survival] | *+/+; UAS-LamB.GFP/+* (n = 120) [87.5% mean survival] | Mann-Whitney | < 0.0001 |
| | 24 h 1% $H_2O_2$ | *Hsp70-Gal4/+; UAS-LamB.GFP/+* (n = 110) [30.9% mean survival] | *+/+; UAS-LamB.GFP/+* (n = 110) [91.8% mean survival] | Unpaired *t*-test with Welch's correction | < 0.0001 |
| | 12 h 10mM PQ | *Hsp70-Gal4/+; UAS-LamB.GFP/+* (n = 130) [63.1% mean survival] | *+/+; UAS-LamB.GFP/+* (n = 139) [94.1% mean survival] | Mann-Whitney | < 0.0001 |
| 3 | PAM DNs imaging [Lam-B/ Hoechst] | Day 10 $w^{1118}$ brain (n= 20 brains) [Mean = 1.07] | Day 30 $w^{1118}$ brain (n= 21 brains) [Mean = 0.97] | Mann-Whitney | n.s. |

| Figure | Type of Assay | Condition 1 (Sample size) [Mean/Median] | Condition 2 (Sample size) [Mean/Median] | Statistical Test | *p*-value |
|---|---|---|---|---|---|
| 4B | Climbing | *R58E02-Gal4/ UAS-LamB.GFP* (n =261) [Mean = 12.39 cm] | *+/UAS-LamB.GFP* (n = 308) [Mean = 9.88 cm] | Mann-Whitney | < 0.0001 |
| 4C | 8 h Dry starvation | *R58E02-Gal4/ UAS-LamB.GFP* (n = 88) [48.1% mean survival] | *+/UAS-LamB.GFP* (n = 91) [46% mean survival] | Unpaired *t*-test | n.s. |
| | 24 h Wet starvation | *R58E02-Gal4/ UAS-LamB.GFP* (n = 100) [73% mean survival] | *+/UAS-LamB.GFP* (n = 95) [89.8% mean survival] | Mann-Whitney | n.s. |
| | 24 h 1% $H_2O_2$ | *R58E02-Gal4/ UAS-LamB.GFP* (n = 100) [87% mean survival] | *+/UAS-LamB.GFP* (n = 100) [98% mean survival] | Mann-Whitney | <0.02 |
| 4D | Lifespan | *R58E02-Gal4/ UAS-LamB.GFP* (n = 150) [Median = 58.5 days] | *+/UAS-LamB.GFP* (n = 142) [Median = 62 days] | Log-rank | n.s. |
| 5B | Level of 100 kD Lamin-B | Day 10 (6 replicates) [Mean 100 kD Lamin-B band = 1] | Day 30 (6 replicates) [Mean 100 kD Lamin-B band = 3.03] | Paired *t*-test | 0.01 |
| 5D | Solubility of Lamin-B | Day 10 (7 replicates; 100 mg /replicate) [Mean Lamin-B = 1] [Mean CP190 = 1] | Day 30 (7 replicates; 100 mg /replicate) [Mean Lamin-B = 2.55] [Mean CP190 = 0.56] | Paired *t*-test | < 0.04 |
| | Solubility of CP190 | | | Paired *t*-test | < 0.02 |
| S4D | Climbing | *TH-Gal4/ UAS-LamB.GFP* (n = 152) [Mean = 9.01 cm] | *+/UAS-LamB.GFP* (n = 171) [Mean = 10.74 cm] | Mann-Whitney | < 0.0001 |
| S4E | Climbing | *DJ667-Gal4/ UAS-LamB.GFP* (n = 157) [Mean = 5.23 cm] | *+/UAS-LamB.GFP* (n = 197) [Mean = 7.79 cm] | Mann-Whitney | <0.0001 |
| S4F | Lifespan | *Hsp70-Gal4/+; UAS-LamB.GFP/+* (n = 180) [Median = 49 days] | *+/+; UAS-LamB.GFP/+* (n = 164) [Median = 58 days] | Log-rank | < 0.0001 |
| S4G | Lifespan | *TH-Gal4/ UAS-LamB.GFP* (n = 270) [Median = 32 days] | *+/UAS-LamB.GFP* (n = 262) [Median = 54 days] | Log-rank | < 0.0001 |
| S4H | Lifespan | *DJ667-Gal4/ UAS-LamB.GFP* (n = 135) [Median = 47 days] | *+/UAS-LamB.GFP* (n = 132) [Median = 50 days] | Log-rank | 0.0005 |

All Mann-Whitney and Unpaired *t*-tests (with or without Welch's correction) are performed with two-tail distribution.

**Fig.4B & S3C**. Climbing assay analysed with Mann-Whitney test

| ***Genotype*** **(Mean Height/cm)** | *R58E02gal4/ UAS-LamB* (12.39) | *+/UAS-LamB* (9.885) | *R58E02gal4/+* (6.574) |
|---|---|---|---|
| *+/UAS-LamB* (9.885) | p < 0.0001 | | |
| *R58E02gal4/+* (6.574) | p < 0.0001 | p < 0.0001 | |
| *+/+* (5.33) | p < 0.0001 | p < 0.0001 | p < 0.005 |

**Fig.4D.** Lifespan assay analysed with Log-Rank test

| ***Genotype*** **(Median Age/days)** | *R58E02gal4/ UAS-LamB* (58.5) | *+/UAS-LamB* (62) | *R58E02gal4/+* (55) |
|---|---|---|---|
| *+/UAS-LamB* (62) | n.s. | | |
| *R58E02gal4/+* (55) | p < 0.0001 | p < 0.0001 | |
| *+/+* (56) | p < 0.0001 | p < 0.0001 | p = 0.001 |

**Fig.S2B.** Western of Lamin-B/β-tubulin with 2-tailed unpaired t-test (3 replicates of 5 heads)

| **Age (LamB/β-tub ± SD)** | **Day 10 (3.84 ± 0.39)** | **Day 20 (2.39 ± 0.11)** | **Day 30 (1.83 ± 0.26)** |
|---|---|---|---|
| **Day 10 (3.84 ± 0.39)** | | p = 0.003 | p < 0.002 |
| **Day 20 (2.39 ± 0.11)** | p = 0.003 | | p < 0.03 |

**Fig.S2C.** Climbing Assay of female $w^{1118}$ flies with Mann-Whitney test

| **Age, n (Mean Height/cm)** | **Day 10, n=111 (7.028)** | **Day 20, n=101 (5.979)** | **Day 30, n=90 (3.442)** |
|---|---|---|---|
| **Day 20, n=101 (5.979)** | p = 0.0004 | | p < 0.0001 |

**Fig.S2D.** Stress test of female $w^{1118}$ flies. Mann-Whitney (M-W) test.

| **Dry 12 h** | **Day 20** | **Day 30** | **Wet 48 h** | **Day 20** | **Day 30** |
|---|---|---|---|---|---|
| **Day 10** | p < 0.02 (M-W) | p = 0.03 (*t*-test) | **Day 10** | p = 0.03 (*t*-test) | n.s. (*t*-test) |
| **$H_2O_2$ 48 h** | **Day 20** | **Day 30** | **PQ 24 h** | **Day 20** | **Day 30** |
| **Day 10** | p < 0.003 (M-W) | p = 0.001 (M-W) | **Day 10** | p < 0.02 (M-W) | p < 0.02 (M-W) |

**Fig.S3B.** Climbing Assay with Mann-Whitney test

| ***Genotype*** **(Mean Height/cm)** | *Hsp70/+; UAS-LamB/+* (11.45) | *+/+; UAS-LamB/+* (8.871) | *Hsp70/+; +/+* (9.366) |
|---|---|---|---|
| *+/+; UAS-LamB/+* (8.871) | $p < 0.0001$ | | |
| *Hsp70/+; +/+* (9.366) | $p < 0.0001$ | n.s. | |
| *+/+; +/+* (3.944) | $p < 0.0001$ | $p < 0.0001$ | $p < 0.0001$ |

**Fig.S4F.** Lifespan Assay with Log-rank test

| ***Genotype*** **(Median Age/days)** | *Hsp70/+; UAS-LamB/+* (49) | *+/+; UAS-LamB/+* (58) | *Hsp70/+; +/+* (51) |
|---|---|---|---|
| *+/+; UAS-LamB/+* (58) | $p < 0.0001$ | | |
| *Hsp70/+; +/+* (51) | n.s. | $p < 0.0001$ | |
| *+/+; +/+* (54) | n.s. | $p < 0.0001$ | $p < 0.005$ |

## 8) Original uncropped immunoblot images

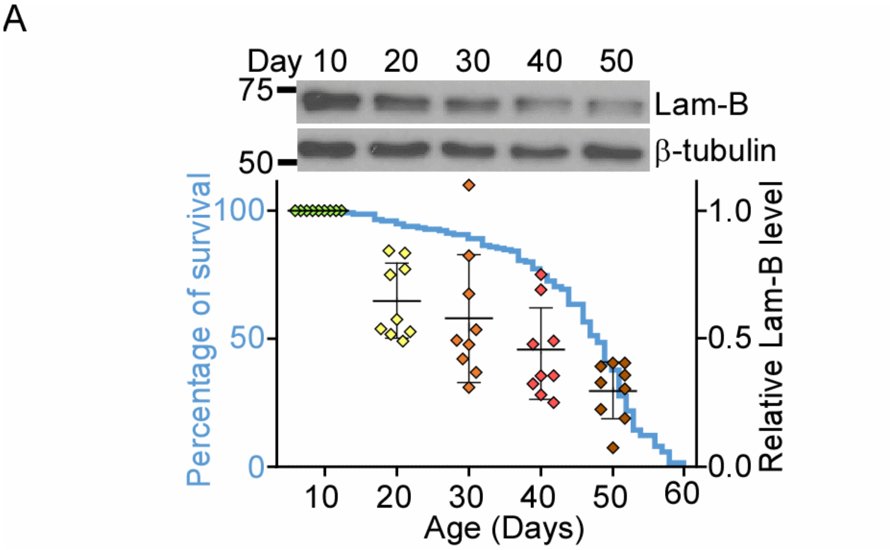


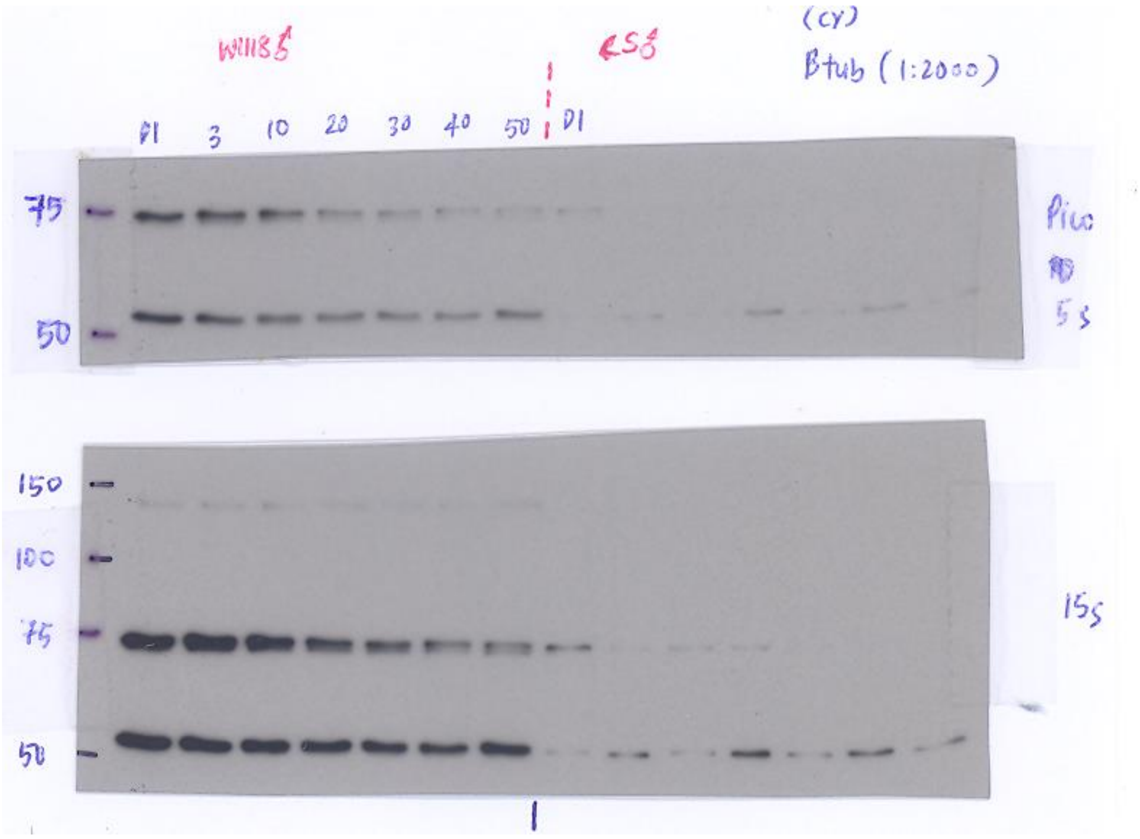

Two different exposure time. Bottom image (from D10 to D50) was used for Figure 1A.

Images acquired on KODAK film.

B

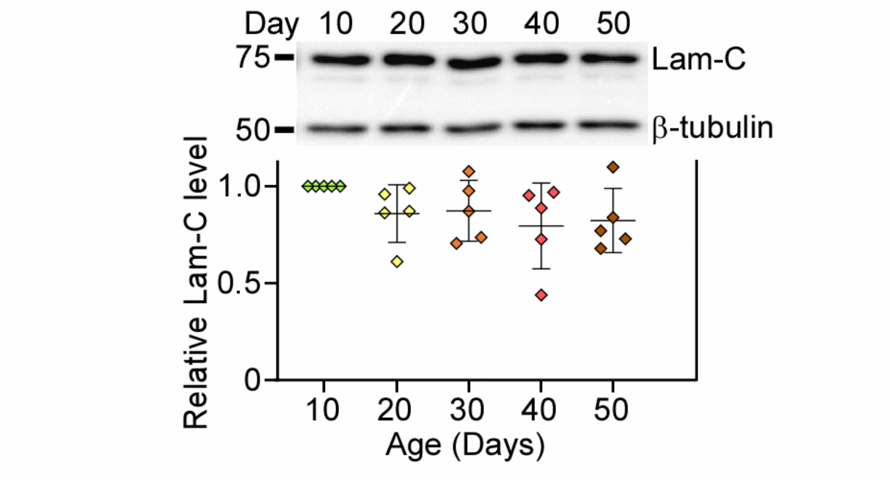


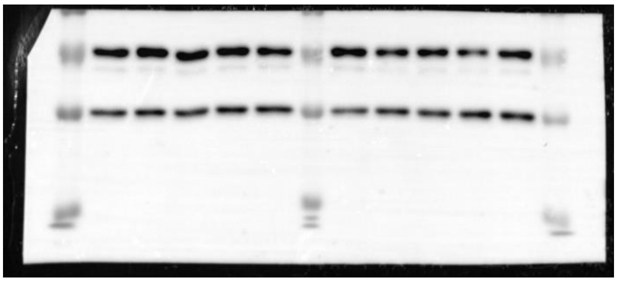

**Left image was used for Figure 1B.** ***BIO-RAD ChemiDoc Imaging System.***

B

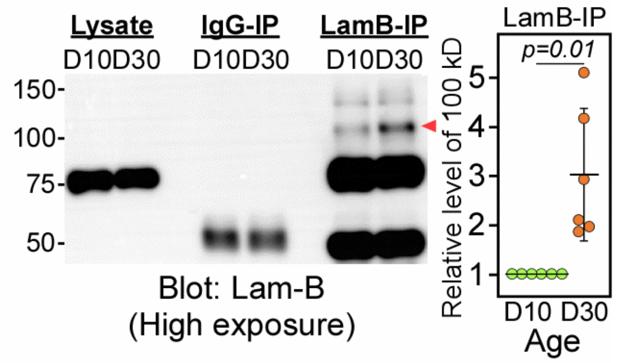
Lysate
IgG-IP
LamB-IP
D10D30
D10D30
D10D30
150-
100-
75-
50-
Blot: Lam-B
(High exposure)
LamB-IP
p=0.01
Relative level of 100 kD
5
4
3
2
1
D10 D30
Age

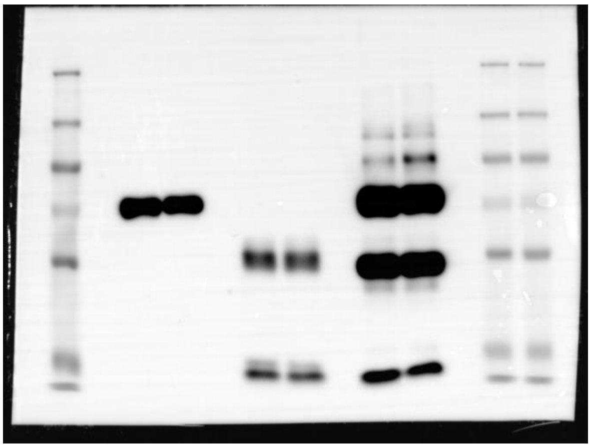

C

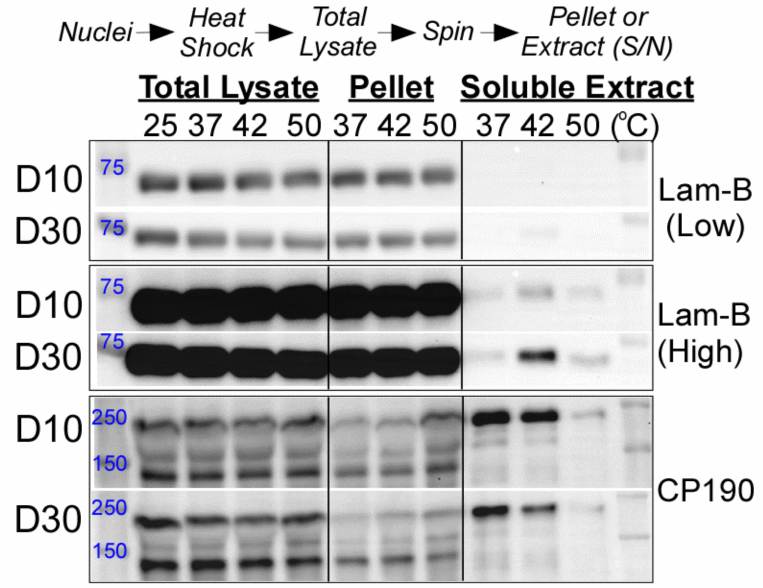


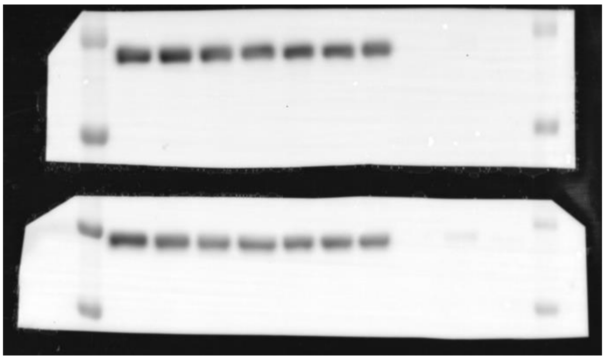

**D10 Lam-B**

**D30 Lam-B**

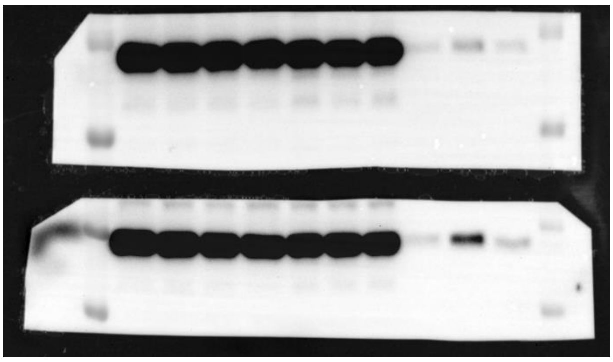

**D10 Lam-B**

**D30 Lam-B**

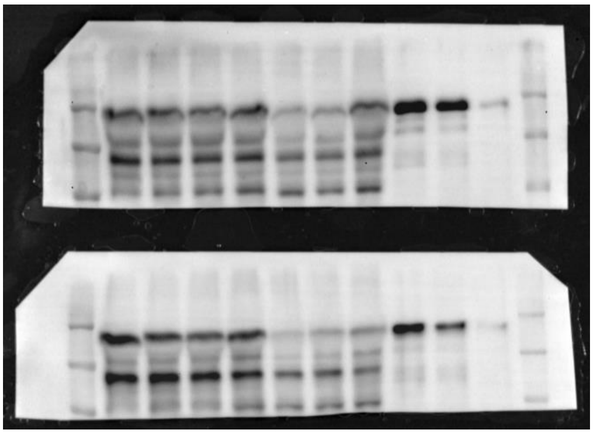

**D10 CP190**

**D30 CP190**

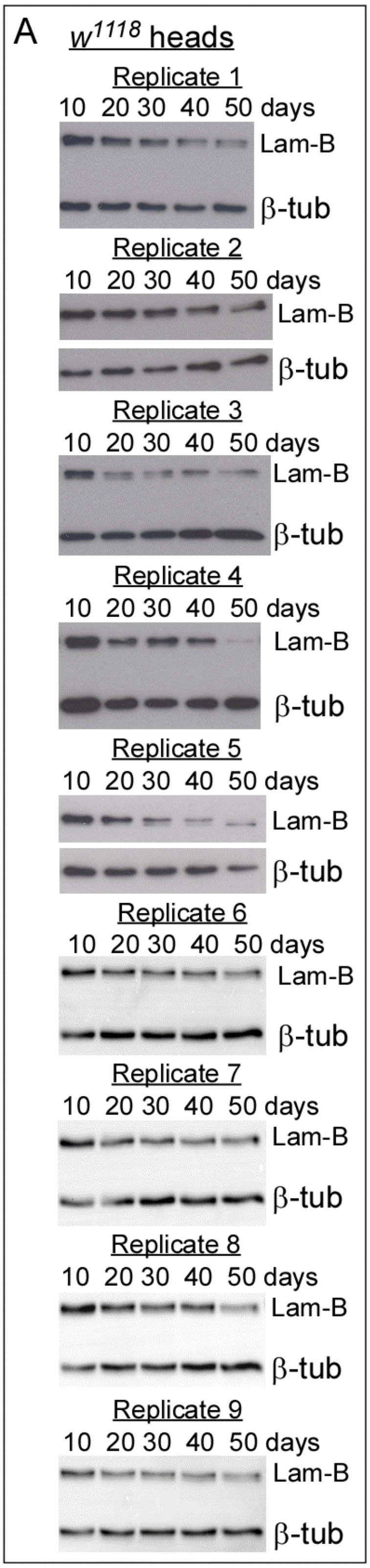


***Figures from Kodak film.***

Replicate 1 is from Figure 1A and has been shown above.

Replicate 2: D1, D3, D10, D20, D30, D40, D50. Only D10-D50 were used.

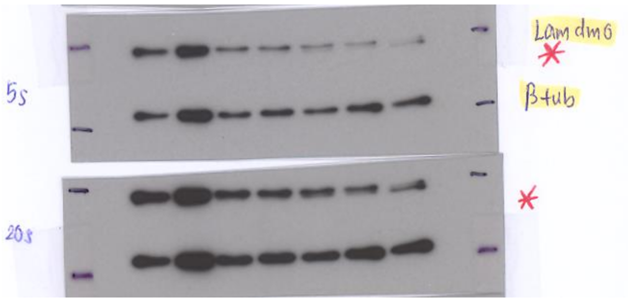


Replicate 3: D1, D3, D10, D20, D30, D40, D50.

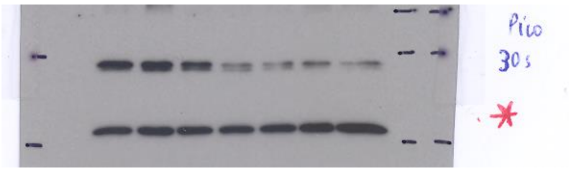


Replicate 4: From left - D1, D3, D10, D20, D30, D40, D50, (**not used:** D1,3,10,20,30,50).

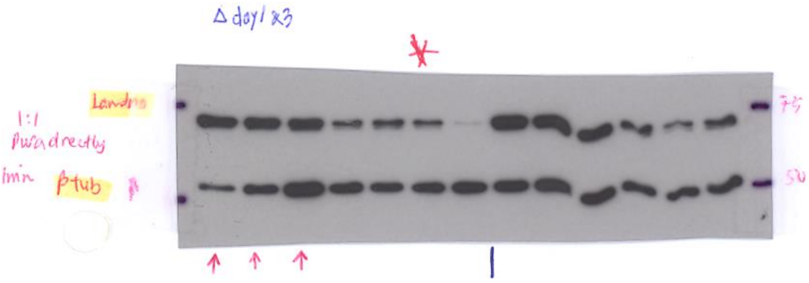


Replicate 5: D1, D10, D20, D30, D40, D50. Different exposure time. * were used.

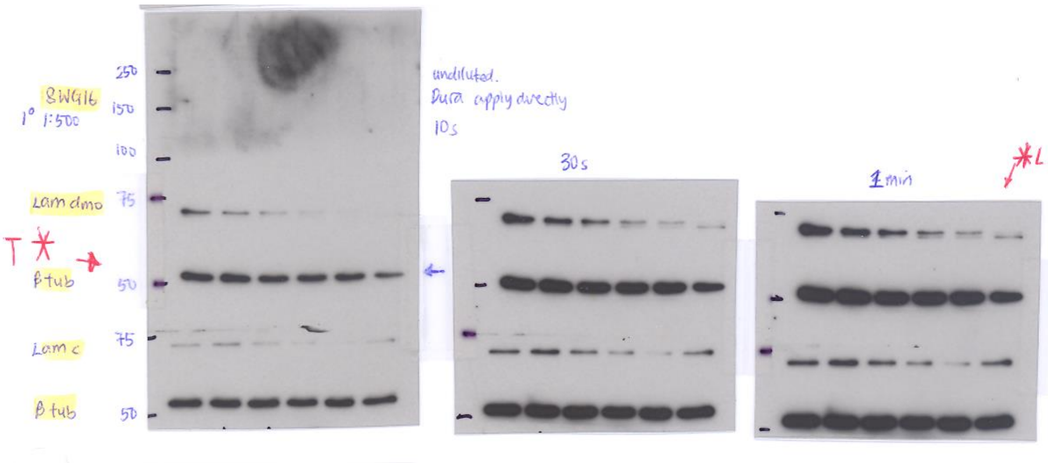

***BIO-RAD ChemiDoc Imaging System.***

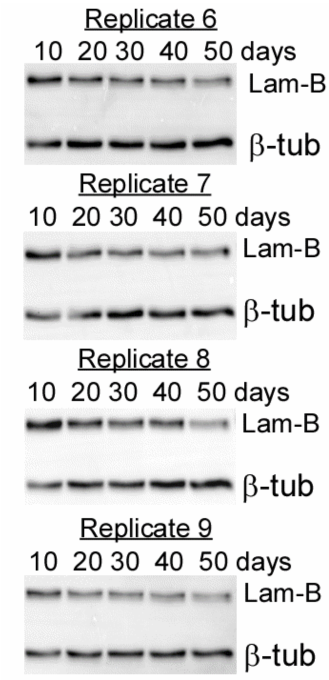


<u>Replicate 6: D1, D3, D10, D20, D30, D40, D50.</u>

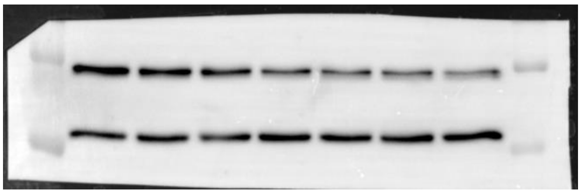

<u>Replicate 7: D1, D3, D10, D20, D30, D40, D50.</u>

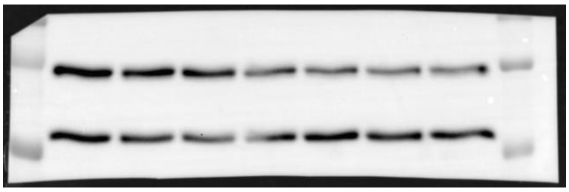

<u>Replicate 8: D1, D3, D10, D20, D30, D40, D50.</u>

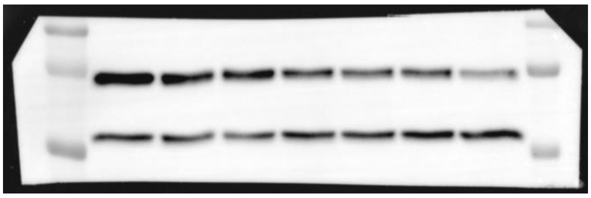

<u>Replicate 9: D1, D3, D10, D20, D30, D40, D50.</u>

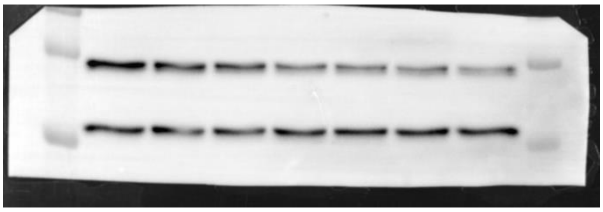

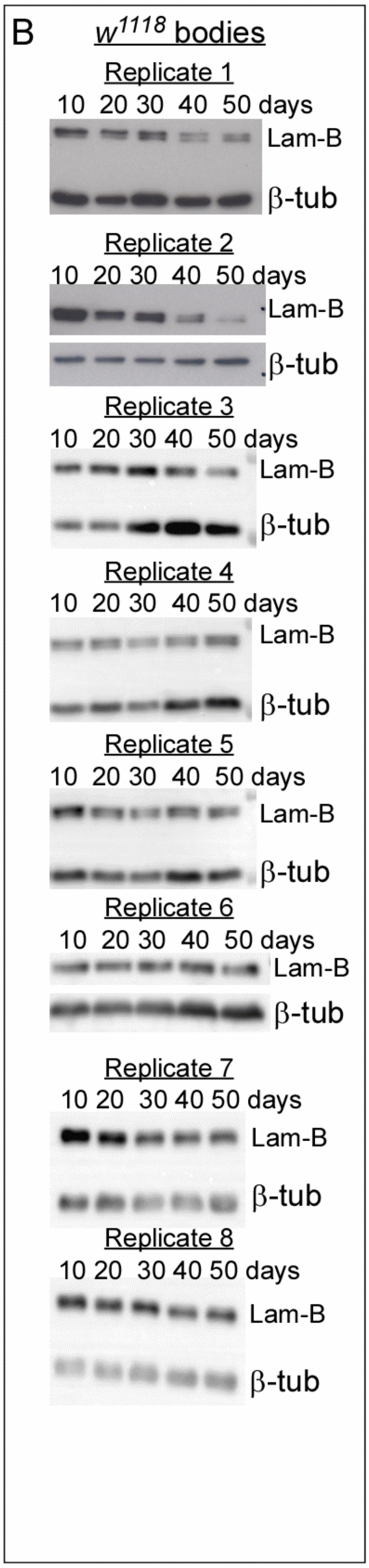


Replicate 1 (three different exposure time. Green asterix was used).

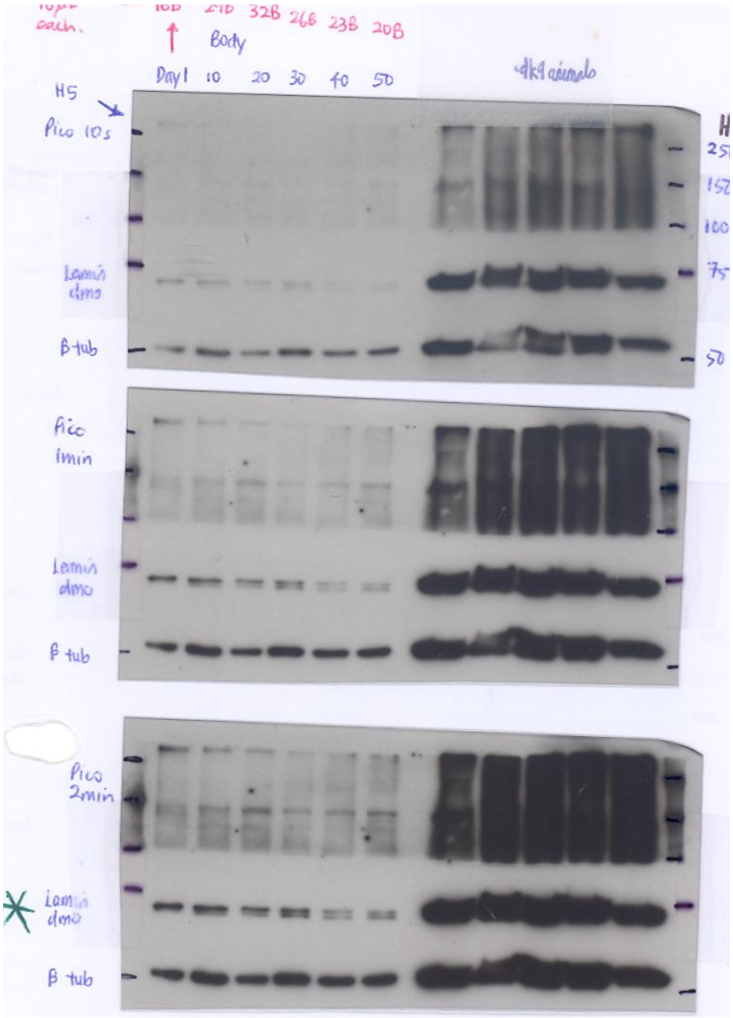

Replicate 2: (Left Panel, red asterix): D1, D10, D20, D30, D40, D50

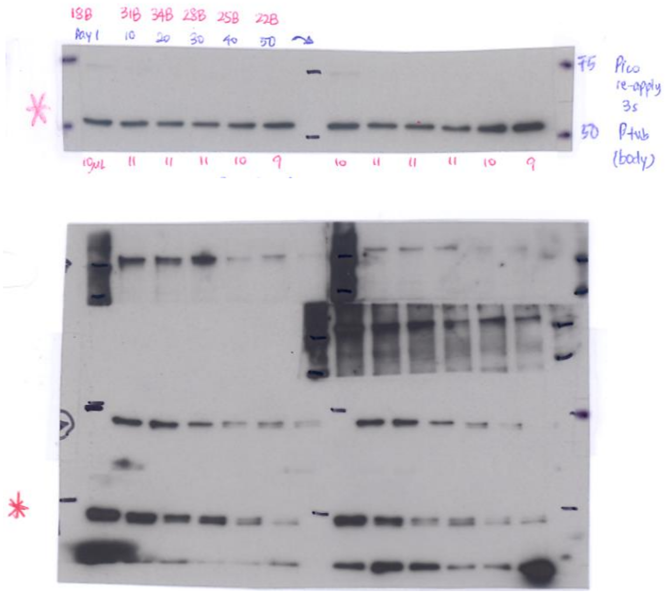

Lam-B

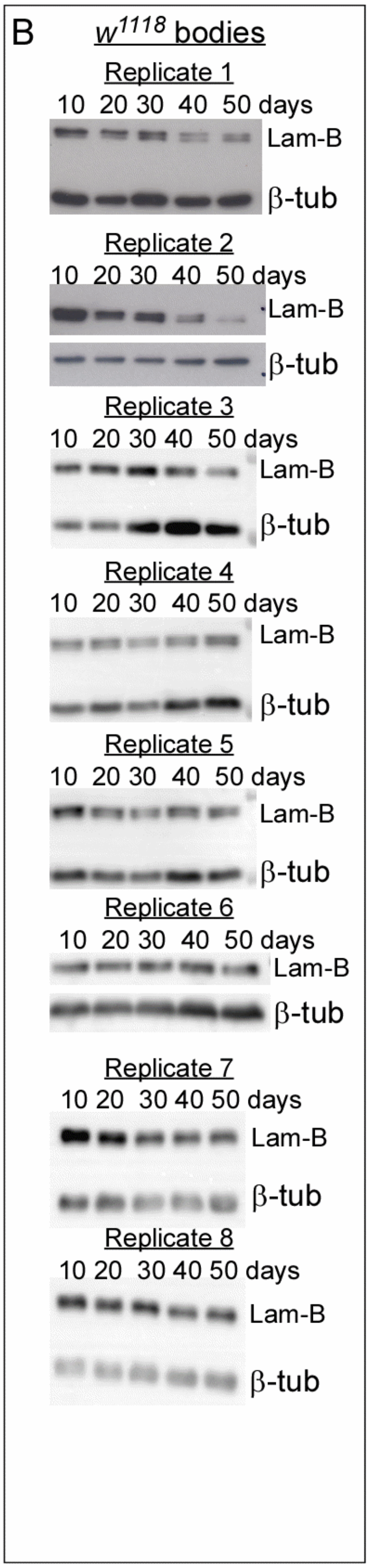


Replicate3: D3, 10, 20, 30, 40, 50, 3 (not used); D10, D20, D30, D40, D50 (used).

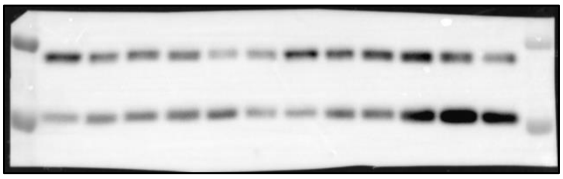

Replicate 4: D3 (not used); D10, D20, D30, D40, D50.

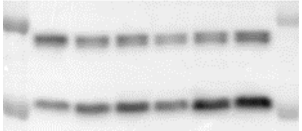

Replicate 5: D3 (not used); D10, D20, D30, D40, D50.

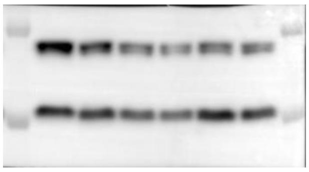

Replicate 6: D1, D3, D10, D20, D30, D40, D50.

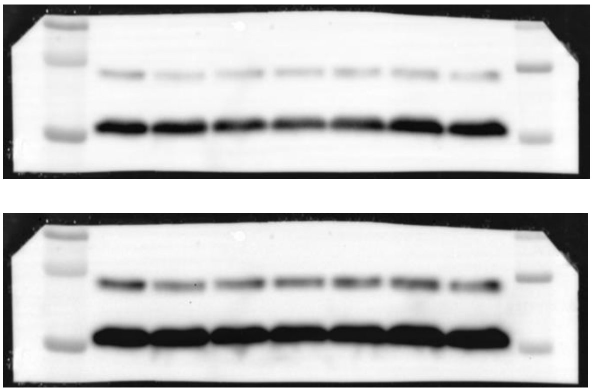

Replicate 7 (left) and Replicate 8 (Right): D10, D20, D30, D40, D50.

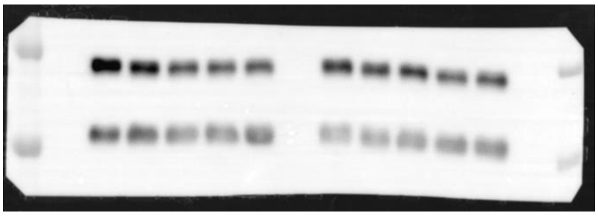

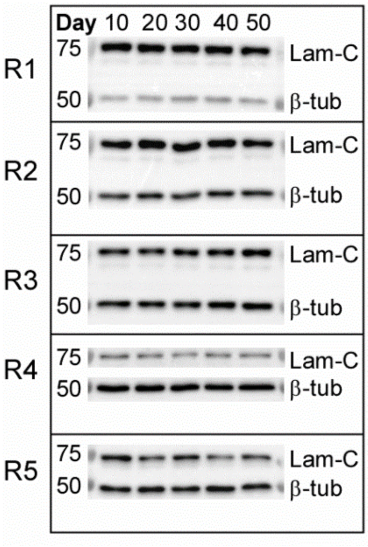


<u>R1</u>

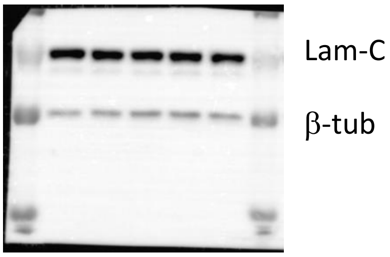


<u>R2</u>

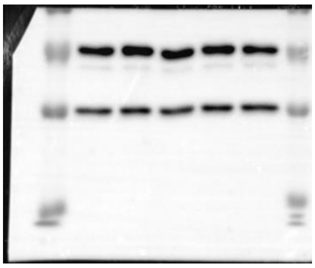

<u>R3</u>

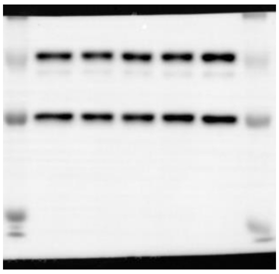

<u>R4</u>

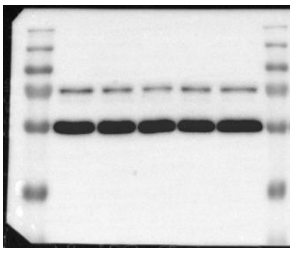

<u>R5</u>

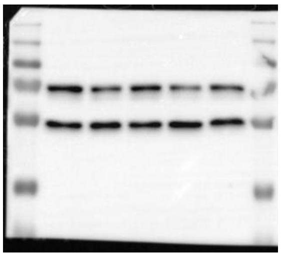

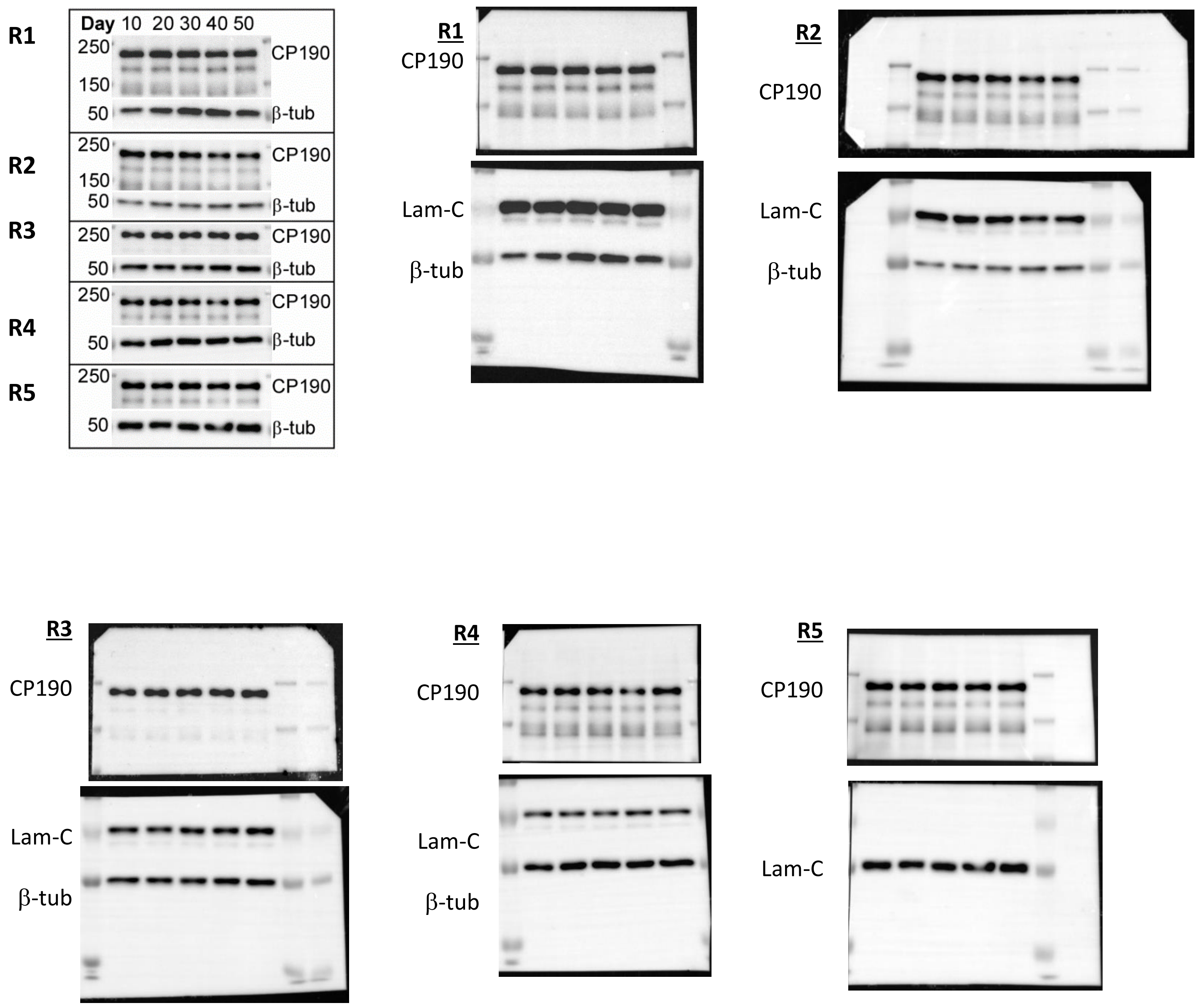
R1
Day 10 20 30 40 50
250
150
50
CP190
β-tub
R2
250
150
50
CP190
β-tub
R3
250
50
CP190
β-tub
R4
250
50
CP190
β-tub
R5
250
50
CP190
β-tub
R1
CP190
Lam-C
β-tub
R2
CP190
Lam-C
β-tub
R3
CP190
Lam-C
β-tub
R4
CP190
Lam-C
β-tub
R5
CP190
Lam-C

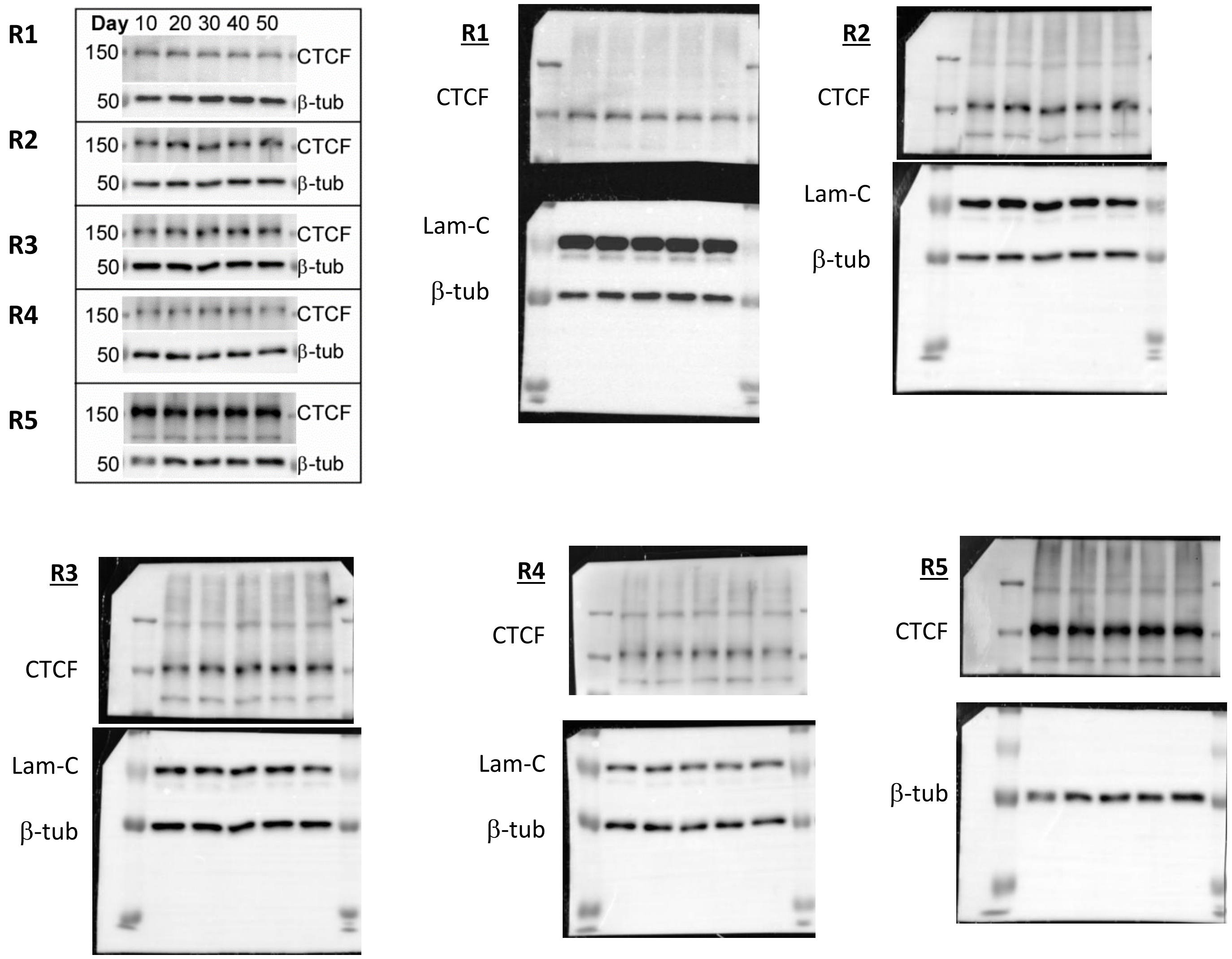
R1
R2
R3
R4
R5
Day 10 20 30 40 50
150
50
CTCF
β-tub
Lam-C

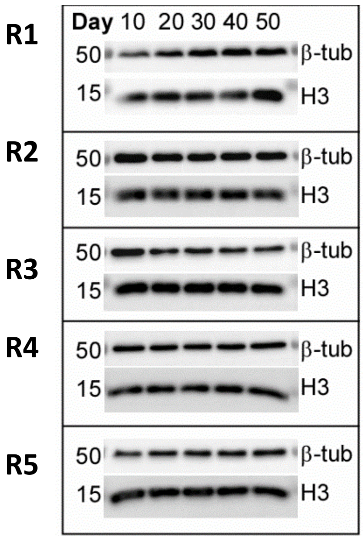


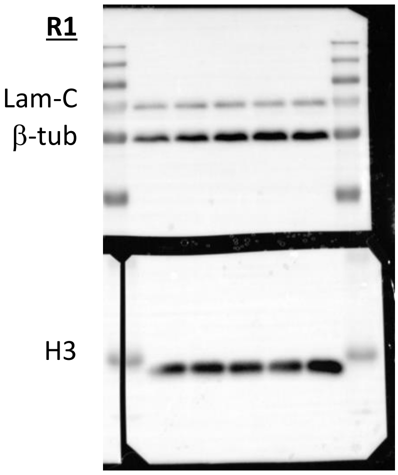


<u>R2 (Top) & R3 (Bottom). Left: β-tub. Right: H3</u>

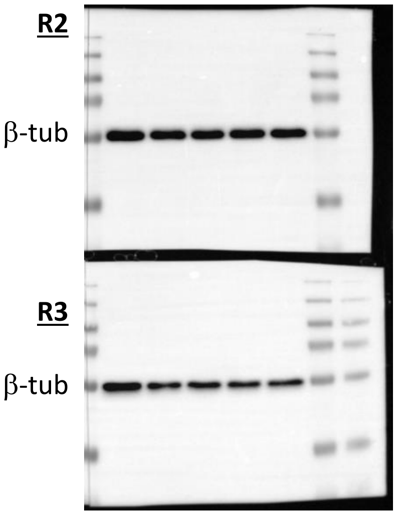


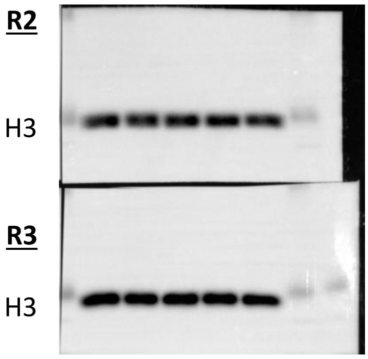


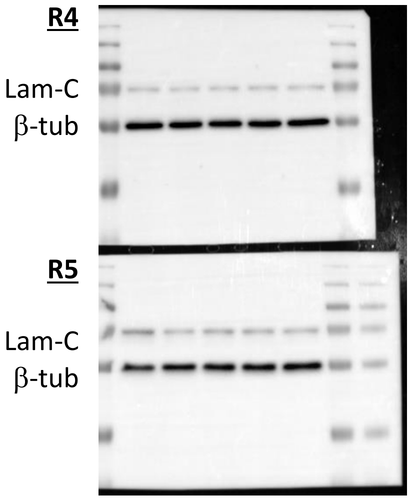


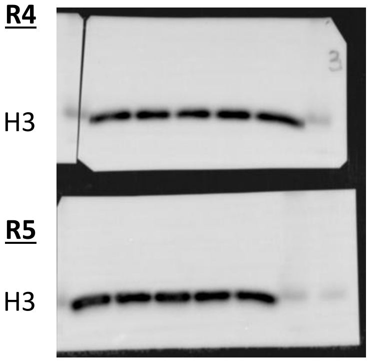

Supplement: S1 File — (PDF) [file pone.0265223.s001.pdf]
