# Supplementary material for: Altered stability of nuclear lamin-B marks the onset of aging in male Drosophila
Source: PLoS One. 2022 Mar 24;17(3):e0265223. doi: 10.1371/journal.pone.0265223 (PMC8947137; doi:10.1371/journal.pone.0265223)
Supplement: Attachment — Submitted filename: Response to Reviewers.pdf [file pone.0265223.s002.pdf]

Response to Reviewers

Reviewer #1: *This a very well written and carefully conducted study showing a possibly important role of lamin-B in the onset of aging in Drosophila. The data have been collected and presented in a very solid and logical manner. The figures are nicely designed and informative. The text is balanced and the conclusions are correct without overinterpretation.*
Minor comments:
- FigS2A: no clear motivation is provided about why specifically the proteins CP190, CTCF and Histone 3 were chosen.
We have added the following sentence: "…several other nuclear proteins that are involved in genome organization. These include chromatin packaging histone H3 protein as well as nuclear architectural proteins like CP190 and CTCF."

- p12, line 12 (results section): pupae lethality > pupal lethality
Thank you, we have made the correction.

- p14, line 26 (discussion section): mechanisms > the mechanisms; remains > remain
Thank you, we have made the correction.

Reviewer #2: *The authors demonstrated that the amount of Lamin-B protein content decreases upon aging in Drosophila adult males., contrarily to other proteins involved in nuclear organization of chromatin like CP190. This suggested a specific role of Lamin-B in the decline of neuronal and physiological functions during aging. The authors tested whether increasing the amount of Lamin-B could improve the functional decline, as well as modify the lifespan of adults. Using ubiquitous expression with the leakage expression level of a hsp70-Gal4 line, they authors show a rescue of the flies performance in the climbing assay, but no rescue of strong stresses like H2O2, paraquat…(there even is a decrease in survival). Building on these data, they further show that increasing the level of expression of Lamin-B in specific dopaminergic neurons is responsible for the climbing assay rescue, and this does affect neither stress responses, nor lifespan.*
To be able to further build on these results, I have several points I would like to be addressed:

- The study seems to have used male flies only. If this is the case, this should appear in the title (in Drosophila males), or I recommend 1° to precise, for each experiment, whether male flies or a mix of male and female flies was used, 2° to verify the obtained results, at least the main ones, with female flies. I can understand that using female bodies can lead to increased variability due to the egg content. However, this is not the case when working with female heads.

First, we have previously conducted all the experiments in male flies. This was written in Materials and methods in first submission. We have since highlighted "male" in the title and throughout the manuscript.

Second, we agreed with reviewer suggestion on performing one set of experiment in female w^{1118} flies. The experiments include measuring the level of lamin-B, climbing ability and stress responses in Day 10, Day 20, and Day 30 female flies. As shown in the new S2B to S2D Fig (Supporting Information, page 4), indeed, the level of lamin-B, climbing ability and most of the stress responses decline in Day 20 female flies. However, Day 10 female flies succumbed to wet starvation more than Day 20 flies. As a result, we focused our studies on male flies.

- In Fig. 2B, there is no decrease of Lam-B content at day 20, but there seems to be a decrease in the content of Lam-B GFP. Because the authors discuss about differences in solubility of the Lamin-B (and no change in the amount of expressed protein) during aging, it would be very interesting to see whether this is also the case for transgenic Lam-B GFP, which can easily be distinguished from Lam-B in western blots.

To address the first point, there was no change in endogenous Lam-B in the previous version of Fig.2B as it was performed using whole body lysates. This is consistent with our findings that Lam-B in the body only starts to decline in Day 30 male fly bodies. Secondly, as overexpression of Lamin-B affects the trajectory of animal physiology and aging, it will be difficult to determine the exact time-point where Lam-B-GFP solubility will be affected.

- Also, the authors do not present the amount of Lam-B GFP in UAS-lam-B GFP/+ flies. We know that UAS promoter can also be leaky depending on the genomic insertion site, and it is possible that there is already expression of Lam-B GFP in this genotype, in particular, considering the rescue activity presented in Fig. S2 and Fig. S3. Western blots should be shown about this (The GFP fluorescence presented in Fig. 2A is not enough, because it is not possible to detect low levels of fluorescence).

We agreed with the reviewer and replaced the previous Fig.2B with a newer version that contains multiple biological replicates as proper controls. From western analysis, there is no expression of LamB-GFP transgene from *UAS-Lam-B-GFP/+ flies* in the absence or presence of heat-shock treatment (Fig 2B). This result rules out the possibility of leaky expression from *UAS-LamB-GFP* transgene in the *UAS-LamB-GFP/+* flies.

On the other hand, in *HSp70-Gal4/UAS-LamB-GFP* flies, we could detect the presence of LamB-GFP protein without heat-shock treatment. This indicates that Hsp70-Gal4 driver is leaky (Fig 2B).

- Also, to confirm 1) that Lam-B-GFP behaves like endogenous Lam-B (same localization into protein complexes, same behavior during aging), the authors could take advantage of the UAS-Lam-B-GFP line (w/o any hsp70 or other Gal4 line) to test for solubility of this protein at different temperatures like in Fig 5C. 2) This would also strengthen their data about changes in solubility (or PTM / or interactome) of Lam-B during aging.

This experiment is not possible as *UAS-Lam-B-GFP* line is not leaky and no Lam-B-GFP protein is expressed in the absence of *Gal4* driver.

To confirm if Lam-B-GFP behaves like endogenous Lam-B, we instead performed a nuclear extract experiment from *HSp70-Gal4/UAS-LamB-GFP* flies. As shown in Fig 2C, both LamB-GFP and endogenous LamB are enriched in nuclear fraction but not in cytoplasm. This suggests that LamB-GFP exhibits similar subcellular localization pattern as endogenous lamin-B.

- What are the data for +/+ flies or hsp70-Gal4/+ flies in the stress experiments in the conditions tested in Fig. 2 (8h, 12h and 24h?).

We have performed the different stress tests for +/+ and *Hsp70-Gal4/+* flies. The results are presented in S3C Fig (Supporting Information, page 6). The results showed that only *Hsp70-Gal4/UAS-LamB-GFP* flies have lower stress resistance whereas all three control lines have similar patterns.

- In all lifespan data presented, the longest lifespan is for UAS-LamB-GFP, suggesting again some leakage of expression from this stock (see point 3). Testing this hypothesis would allow a better interpretation of the author's results.

We are unable to explain why *UAS-LamB-GFP/+* has much longer lifespan than *Hsp70-Gal4/UAS-LamB-GFP*, *TH-Gal4/UAS-Lam-GFP* and *DJ667-Gal4/UAS-LamB-GFP* lines. Strikingly, *R58E02-Gal4/UAS-LamB-GFP* line has similar lifespan as *UAS-LamB-GFP/+ line*. These results highlighted that it is important to drive tissue-specific expression of LamB-GFP so as not to compromise other aspect of animal healthspan.

Furthermore, consistent with the absence of leaky expression from LamB-GFP transgene, the *UAS-LamB-GFP/+* line has lower locomotor activity (S3B Fig) and similar stress resistance profile as other control lines (S3C Fig). Taken together, these results support our interpretation that global ectopic expression of LamB-GFP may improve locomotor activity at the expense of stress resistance and longevity in male flies.
